# S44. LUMATEPERONE (ITI-007) FOR THE TREATMENT OF SCHIZOPHRENIA: PLACEBO-CONTROLLED CLINICAL TRIALS AND AN OPEN-LABEL SAFETY SWITCHING STUDY

**DOI:** 10.1093/schbul/sby018.831

**Published:** 2018-04-01

**Authors:** Kimberly Vanover, Alex Dmitrienko, Steven Glass, Susan Kozauer, Jelena Saillard, Michal Weingart, Andrew Satlin, Sharon Mates, Christoph Correll, Robert Davis

**Affiliations:** 1 Intra-Cellular Therapies, Inc; 2 Mediana Research; 3 The Zucker Hillside Hospital, Hofstra North Shore LIJ School of Medicine

## Abstract

**Background:**

Lumateperone (ITI-007) is a first-in-class investigational agent in development for the treatment of schizophrenia. Acting synergistically through serotonergic, dopaminergic and glutamatergic systems, lumateperone represents a new approach to the treatment of schizophrenia and other neuropsychiatric disorders. Lumateperone is a potent antagonist at 5-HT2A receptors and exhibits serotonin reuptake inhibition. Lumateperone also binds to dopamine D1 and D2 receptors acting as a mesolimbic/mesocortical dopamine phosphoprotein modulator (DPPM) with pre-synaptic partial agonism and post-synaptic antagonism at D2 receptors and as an indirect glutamatergic (GluN2B) phosphoprotein modulator with D1-dependent enhancement of both NMDA and AMPA currents via the mTOR protein pathway.

**Methods:**

Lumateperone was evaluated in 3 controlled clinical trials to evaluate efficacy in patients with acute schizophrenia. In Study ITI-007-005, 335 patients were randomized equally across 4 treatment arms: one of two doses of lumateperone, risperidone (active control) or placebo QAM for 4 weeks. In Study ITI-007-301, 450 patients were randomized equally across 3 treatment arms: one of two doses of lumateperone or placebo QAM for 4 weeks. In Study ITI-007-302, 696 patients were randomized equally across 4 treatment arms: one of two doses of lumateperone, risperidone (active control) or placebo QAM for 6 weeks. In all 3 studies, the primary endpoint was change from baseline on the Positive and Negative Syndrome Scale (PANSS) total score compared to placebo. Also, a 6-week open-label safety switching study was conducted. In this ITI-007-303 study 302 patients with stable schizophrenia were switched from standard-of-care (SOC) antipsychotics and treated for 6 weeks with lumateperone QPM outpatient and then switched back to SOC for 2 weeks.

**Results:**

Two of the 3 randomized studies were positive. In Studies ITI-007-005 and ITI-007-301, lumateperone (60 mg ITI-007, equivalent to 42 mg active base) met the primary endpoint with statistically significant superior efficacy over placebo at Day 28 as measured by the PANSS total score (Study ITI-007-005 p=0.017; Study ITI-007-301 p=0.022). In Study ITI-007-302, neither dose of lumateperone separated from placebo on the primary endpoint in the intent-to-treat population; a high placebo response was observed in this study. Across all 3 efficacy trials, lumateperone improved symptoms of schizophrenia with the same trajectory and same magnitude of improvement from baseline on the PANSS total score.

Lumateperone was well-tolerated with a favorable safety profile in all studies. In the two studies with risperidone included as an active control, lumateperone was statistically significantly better than risperidone on key safety and tolerability measures including prolactin, glucose, lipids and weight. In the open-label safety switching study statistically significant improvements from SOC were observed in body weight, cardiometabolic and endocrine parameters worsened again when switched back to SOC medication. In this open-label study, symptoms of schizophrenia generally remained stable or improved. Greater improvements were observed in subgroups of patients with elevated symptomatology such as those with comorbid symptoms of depression and those with prominent negative symptoms.

**Discussion:**

Lumateperone represents a novel approach to the treatment of schizophrenia with a favorable safety profile in clinical trials. The lack of metabolic, motor and cardiovascular safety issues presents a safety profile differentiated from standard-of-care antipsychotic therapy.

